# *Nigella sativa* and its nano‐mediated approach toward management of neurodegenerative disorders: A review

**DOI:** 10.1002/ibra.12091

**Published:** 2023-02-07

**Authors:** Chaitali G. Gawas, Sakshi Mathur, Minal Wani, Heena Tabassum

**Affiliations:** ^1^ Dr. D. Y. Patil Biotechnology and Bioinformatics Institute, Dr. D. Y. Patil Vidyapeeth Pune Maharashtra India

**Keywords:** nanoparticles, nanotechnology, neurodegenerative diseases, *Nigella sativa*, thymoquinone

## Abstract

*Nigella sativa* L., also known as black seed or black cumin, is a plant that has been used for centuries. In the past, this flowering plant was used as a food preservative and medicinal herb. A vital component of *Nigella sativa*, thymoquinone (TQ), plays a significant therapeutic role in the management of most diseases, including cancer, diabetes mellitus, hypertension, inflammation, gastrointestinal disorders, and neurodegenerative disorders. Neurodegenerative disorders are primarily caused by neurotransmitter hypoactivity, particularly insufficient serotonin activity. It has been discovered that many medicinal herbs and their active compounds have therapeutic value. Black cumin seeds have been used to heal ailments and its history traces back to ancient times such as ancient Babylonia. They can be used applied to alleviate edema, hair loss, and bruising, and consumd to treat stomach issues. It is one of the most feasible and effective medicinal plants. The use of nanoformulations based on *Nigella sativa* and TQ to treat neurodegenerative diseases (NDs) has yielded promising outcomes. Customized administration of nanoparticle (NP) systems and nanomedicine are two of the many options for drug delivery to the central nervous system (CNS) that are attracting increasing interest. Delivering a therapeutic and diagnostic substance to a particular location is the core target of NPs. Because of their distinct cell uptake and trafficking mechanisms, NPs can reduce the amount that accumulates in undesirable organs. The focus of the current review is on recent studies on the various neuroprotective properties of *Nigella sativa* as well as nanoformulations for NDs and the brain's uptake of NPs. The review summarizes the In vivo, In vitro, and In silico studies on the protective effects of black cumin against neurodegenerative disorders.

## Graphical abstract

*Nigella sativa* is a flowering plant that grows annually. So far, numerous studies have shown that the *Nigella sativa* seed and its primary active ingredient, thymoquinone (TQ), are extremely effective medicines for a variety of conditions, including neuroprotective and antioxidant properties. Epilepsy is one of the most prevalent diseases in the world, along with other neurological disorders. Such disorders can be treated with *Nigella sativa* nanoformulations targeting the central nervous system. Current nanoformulations include TQ rich fraction nanoemulsion, TQ solid‐lipid nanoparticles (NPs), TQ‐loaded poly(lactic‐co‐glycolic acid) chitosan NPs, and TQ mucoadhesive nanoemulsions.

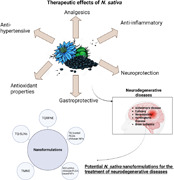

## 1 INTRODUCTION

The range of brain disorders is broad and includes many conditions that are listed in the chapters on neurological or mental disorders of well‐established international diagnostic classification systems. Both short‐ and long‐term impairments and disabilities are common among these disorders. Consequently, they place a strain on the patients, their families, and their social network at emotional, financial, and social levels. Serious disorders like Alzheimer's disease (AD), Parkinson's disease (PD), Lewy body dementia, frontotemporal dementia, vascular dementia, and some uncommon conditions like amyotrophic lateral sclerosis, Huntington disease, spinocerebellar ataxia, and prion diseases are included in the category of neurodegenerative diseases (NDs).^1^ Despite significant clinical differences, neurodegenerative disorders share common features such as late onset, extensive neuronal loss, and synaptic abnormalities, and the presence of misfolded protein aggregates in the brain.^2^ For a very long time, herbal remedies or traditional medicines were frequently used to treat a variety of diseases in a number of different countries (both developed or developing). The data showed a natural drug preference in hypothetical cases involving a broad range of health conditions, as well as a mild vs. a severe specific health condition (hypertension). It is possible that this preference is also observable in circumstances where death is imminent such as those involving cancer, cardiovascular disease, or serious infections. It will be instructive to explore this option further through research.^3^ As cancer chemotherapeutic agents, many plant‐based products have successfully entered the market. Herbal therapy is a holistic treatment that addresses emotional, mental, and spiritual levels. Any naturopathic method takes lifestyle, behavioral, and cognitive issues into account. Since medicinal plants are widely believed to be safe, using herbs typically does not result in drug behaviours or negative effects. Furthermore, informed knowledge of the effects of medicinal plants is required, as is conducting a clinical trial, to determine the appropriate medical application.^4^ In a recent study by Welz et al.^5^ the most frequently cited reasons why herbal medicine was preferred as a form of treatment included dissatisfaction with conventional therapy, prior positive experiences, positive aspects associated with herbal medicine, and family traditions. By contrast, synthetic drugs are created in a lab using a variety of methodologies, and these are medications that are not found in nature. Synthetic drugs exert severe negative side effects on the human body in addition to curing diseases. There are multiple examples in the literature that report on the negative effects of synthetic drugs; for example, even though paracetamol is a commonly used antipyretic, one of its severe side effects is liver poisoning. In some cases, herbal remedies are less potent than synthetic ones, but they are still regarded as less toxic or as having fewer side effects than synthetic ones. The most important standards for all medications, whether they are made naturally or artificially, are potency, stability, specificity, and nontoxicity. There are numerous examples in the literature on the negative effects of synthetic drugs; on example is the well‐known antipyretic drug paracetamol, which can also severely harm the liver. Spices like turmeric, cloves, cinnamon, and chilies, in addition to herbs, may have some therapeutic effects on human health.^6^ For instance, turmeric contains curcumin, which has therapeutic properties such as its anti‐inflammatory activity, antiplatelet activity, hepatoprotective action, and its anticancer activity.^7^ Instead of searching for synthetic drugs to treat any disease, use of natural medicines such as plant polyphenols, alkaloids, and tannins can decrease the side effects and toxicities of their synthetic counterparts while maximizing therapeutic outcomes with the most potent and dynamic healing effects. It was also discovered that use of plant derivatives and chemicals could be an important strategy for preventing or delaying neurological deterioration in dementia patients.^8^ The therapeutic applications of pharmaceutical substances have grown significantly in the last few decades. This is primarily due to their acceptable efficacy and fewer side effects. Drugs to improve cognition are becoming more necessary as the ones currently on the market, such as psychostimulants, have the potential to be abused and can cause hallucinations. In this regard, the use of conventional natural substances for treating anxiety and improving cognition is becoming more popular.^9^ Therefore, nanoformulations based on *Nigella sativa (N. sativa)* and thymoquinone (TQ) that target the central nervous system (CNS) and have few side effects have shown promising results in the treatment of NDs.

## 2 ROLE OF NANODRUG DELIVERY IN NEURODEGENERATIVE DISORDERS

Controlling or manipulating substances or objects that are created on a nanometer (one billionth of a meter) scale is referred to as “nanotechnology.” The multifunctional and adaptable architectures of Nanoparticles (NPs) make them suitable for delivery of nanodrugs such as macugen and somavert to the brain. Before they can be used, though, a number of factors must be taken into account, including surface chemistry, hydrophobicity, shape, size, and charge, to name a few. One of the biggest obstacles in CNS‐targeted therapies is crossing of the blood–brain barrier (BBB), and nanotechnology‐based drug delivery systems are crucial in addressing this issue. The BBB acts as an obstacle that keeps out various propagated molecules, along with deadly ones, so that only the right ratio of nutrients passes through instead of harmful/foreign substances. It is possible to create drug delivery systems using nanotechnology that interact with specific cellular subsets or molecules, providing more targeted treatment. With these systems, it is also possible to simultaneously achieve multifunctional qualities like bioactivity, targeting, imaging capabilities, and gene delivery. In this regard, the use of drug delivery methods based on nanotechnology for diagnostic, therapeutic, and rehabilitative purposes has been widely adopted. Numerous studies have been conducted on nanosystems with various biological characteristics and combinations for applications in gene and drug delivery.^10^

Both invasive and noninvasive methods are available for increasing BBB permeability, including intraventricular or intracerebral injections or implantation, infusion, and intranasal administration using drug modification. In some situations, disruption may also work well for crossing of the BBB. The BBB is frequently ruptured to increase the effectiveness of drug delivery to the brain. The physical and chemical properties of NPs determine their path and the methods by which they can cross the BBB. Designing and creating nanoparticles that specifically target the appropriate fraction of affected neurons without damaging other healthy neurons can be tricky. This is crucial, particularly when harmful medications are used to treat aggressive brain tumors.^11^ Transcytosis allows medications or drug‐conjugated NPs to enter the CNS; endocytosis allows pharmaceuticals to cross the BBB.^12^ The mechanisms of receptor‐mediated and adsorptive transcytosis, as well as all of the inherent physicochemical characteristics of neuropharmaceuticals, are being studied by researchers in the neuropharmaceutical field. As a result, improved medicines that can penetrate the BBB might become available.^13^

## 3 NP FORMULATION AS A THERAPEUTIC CHALLENGE FOR NDS

The term “nanoparticle” refers to solid particles or particulate dispersions with sizes between 10 and 1000 nm. The drug is dispersed, trapped, encapsulated, or connected to a matrix of NPs.^14^ These NPs are of interest for medical applications due to their significant and distinctive characteristics, such as their surface to mass ratio, which is significantly higher than that of other particles, their quantum properties, and their capacity to adsorb and transport other substances. Because of their relatively large (functional) surface area, NPs can bind, adsorb, and transport other substances like drugs, probes, and proteins.^15^ Orally administered medications currently used in AD therapy (rivastigmine, galantamine, and donepezil) are efficient for use in patients, but causes side effects because of their activity on peripheral tissues as they lack specificity for their therapeutic targets. For instance, acetylcholinesterase inhibitors can cause nausea and vomiting, which may force patients to stop taking their medication. These challenges could be overcome by creating drug carriers that can pass the BBB and deliver the medication to the CNS at an efficient dose and with a minimal off‐target effects.^16^ Ngowi and colleagues observed that compared to traditional therapy, NPs cause substantially less cerebral poisoning. For instance, an intranasal injection of PLGA NPs is considered to be extremely successful in delivering a mitoNEET ligand antagonist NL‐1 and without any toxicity in a cerebral paradigm. Additional research has revealed that the absorption, bioaccumulation, and prolonged release of cytotoxic agents like amphotericin B, thioridazine, and sorafenib are significantly improved by encapsulation into NPs. Methane and ethane have been demonstrated to be much more soluble in water when silver (Ag) NPs are used, and this dispersion has been found to improve with NPs mass loading. Numerous other potential medications, including astaxanthin, asstilbin, sorafenib, and apigenin, have also been observed to show enhanced absorption and solubility after encapsulation in NPs.^17^

### 3.1 NPs uptake mechanism into the brain

Intravascular administration of NPs represents the typical strategy for achieving a CNS effect. NPs, on the other hand, are rapidly cleared from circulation, resulting in a constrained time in circulation and decreased BBB crossing. It is estimated that the brain can only receive up to 5% of the initial NP dose.^18^

**Figure 1:**
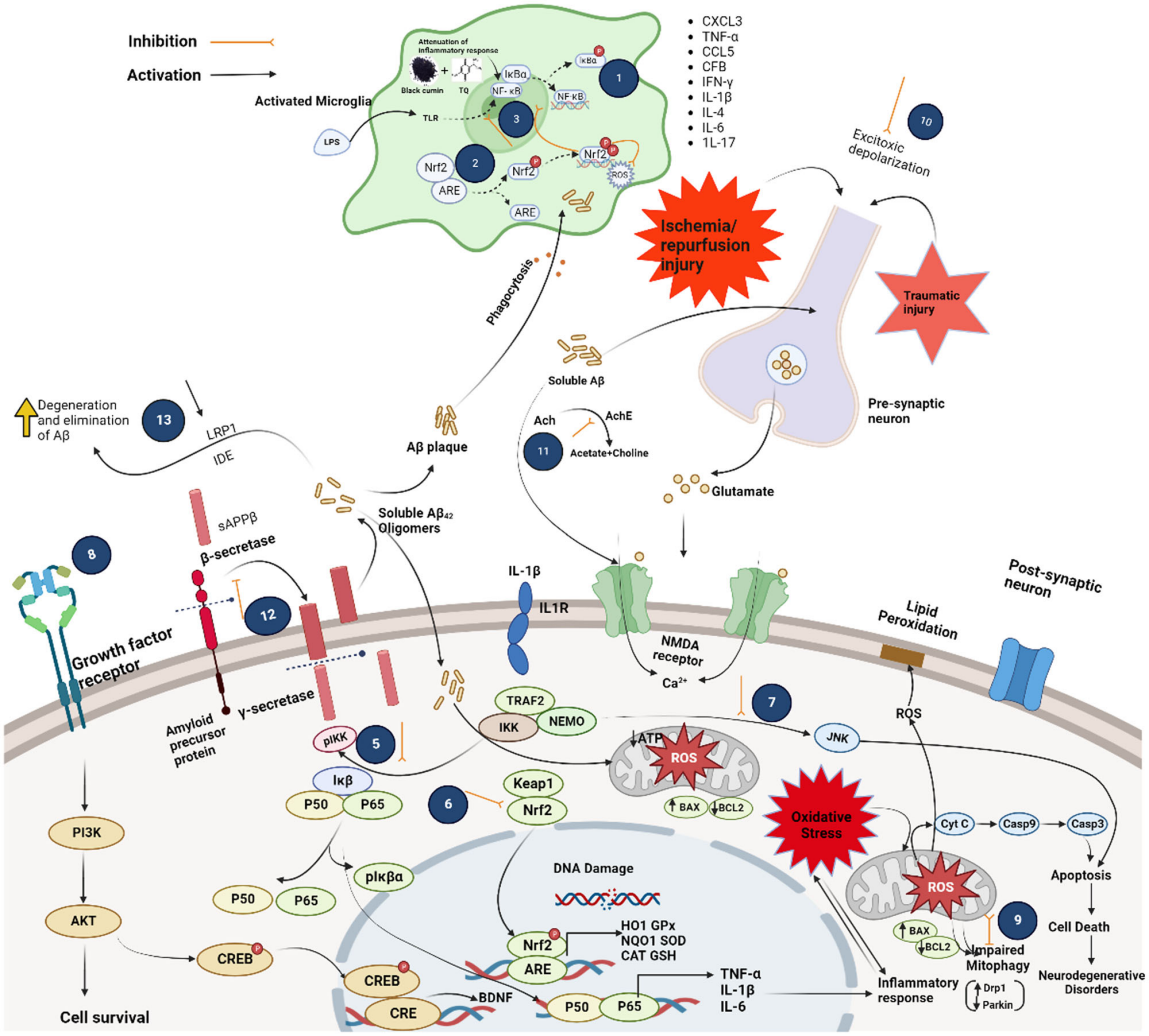
Schematic diagram showing the pathobiology of degenerative brain disorders and the postischemic/traumatic effects of black cumin and thymoquinone (TQ). The neuroprotective mechanisms of black cumin and TQ include the following: (1) inhibition of COX‐2 activity; (2) induction of the antioxidant defense system via activation of the Nrf2/ARE pathway; (3) cross‐talk between Nrf2 and NF‐κB; (4) attenuation of oxidative stress in activated microglia; (5) protection against neuroinflammation by inhibiting NF‐κB signaling; (6) priming of the antioxidant defense system by activation of the Nrf2/ARE pathway; (7) apoptosis prevention via downregulation of the proapoptotic JNK/Erk pathway; (8) initiation of the BDNF‐dependent pro‐survival pathway via induction of PI3K/Akt signaling; (9) recruitment of mitophagy in neurons; (10) attenuation of I/R‐injury by preventing excitotoxic depolarization in the neuron presynaptic terminal; (11) anticholinesterase activity; (12) antiamyloidogenesis via blockage of β‐secretase activity; and (13) clearance of Aβ by upregulating IDE, LRP1, and RAGE. Ach, acetylcholine; AChE, acetylcholinesterase; Akt, protein kinase B; APP, amyloid precursor protein; ARE, antioxidant response element; BDNF, brain‐derived neurotrophic factor; COX2, cyclooxygenase 2; CREB, cAMP‐response element binding protein; Drp1; dynamin‐related protein‐1; GluN2B, *N*‐methyl d‐aspartate receptor subtype 2B; HO‐1, heme oxygenase‐1; IDE, insulin‐degrading enzyme; IkB, NF‐κB inhibitor; IKK, IκB kinase; Keap1, Kelch‐like ECH associated protein 1; IL‐1β, interleukin‐1 beta; IL1R, interleukin‐1 receptor; iNOS, inducible isoform of nitric oxide synthase; JNK, c‐Jun N‐terminal kinases; LPS, lipopolysaccharide; LRP1; low‐density lipoprotein receptor‐related protein 1; NF‐κB (p50‐p65), nuclear factor kappa‐light‐chain‐enhancer of activated B cells; NO, nitric oxide; NQO‐1, NAD(P)H quinone oxidoreductase 1; Nrf2, nuclear factor erythroid 2‐related factor 2; PGE2, prostaglandin E2; PI3K, phosphatidylinositol‐3‐kinase; RAGE, receptor for advanced glycation end‐products; ROS, reactive oxygen species; TLR, toll‐like receptor; ψ, mitochondrial membrane potential.^21^

The cerebral microvessels' lining of brain endothelial cells, which forms the barrier, is used. It is held in place by the astrocytes and pericytes that surround the endothelium. Tight junctions, which tightly connect the endothelial cells together and are one of the endothelium's many barrier‐forming characteristics, are induced in part by these surrounding cells. However, the BBB also acts as a roadblock for transportation. Specific transport proteins and transcytosis mechanisms that control molecule uptake and efflux contribute to the formation of the BBB pathway. The transport of molecules across the barrier can occur in a number of ways. The tight junctions found between brain endothelial cells severely restrict the transport of hydrophilic molecules within cells. Transcellular lipophilic diffusion allows lipid‐soluble molecules with molecular weights below 400 Da to cross the BBB as long as they are not strongly bound to plasma proteins or serve as a substrate for a transport system there. Specific endogenous BBB transporters exist for many molecules that are necessary for brain function, including amino acids, glucose, peptides, and proteins. The brain receives nutrients like glucose, amino acids, and purine bases through carrier‐mediated transcytosis. Only medications such as L‐DOPA, gabapentin, paraquat, and melphalan can be delivered across the BBB that closely resemble the endogenous carrier substrates that will be absorbed because they are substrate selective. At the BBB, receptor binding or adsorption triggers endocytosis. The binding of a receptor‐specific ligand starts the process of receptor‐mediated endocytosis. Following adsorption or binding, the substance is either transcytosed to the plasma membrane or internalized and transported via the early endosome to the lysosome. Endocytosis mediated by receptors or adsorptive processes transports antibodies, lipoproteins, proteins, and NPs into the brain. The BBB also has a large number of efflux mechanisms in addition to the influx systems shown in Figure 1. They aid in the removal of harmful substances from the brain, which is important for reducing the toxic side effects of CNS drug metabolites, and they restrict the entry of molecules into the brain by promoting luminal release of compounds.^19^, ^20^

## 4 ROLE OF *N. SATIVA* IN NEURODEGENERATIVE

### 4.1 Disorders

Black cumin seeds have been used to heal ailments since ancient Babylonia. They can be used physically to alleviate edoema, hair loss, and bruising, and ingested to treat stomach issues.^22^ Many nations have used *N. sativa*'s seeds and their derivatives as a preventative and curative treatment for various diseases, including those in the Middle East, Northern Africa, and Southwest Asia. Alkaloids, saponins, crude fiber, minerals, vitamins, amino acids, proteins, carbohydrates, and 30% fixed oils make up *N*. *sativa*.^23^

Azzubaidi and colleagues proved that *N. sativa* seeds can significantly preserve spatial cognition in rats suffering from chronic cerebral hypoperfusion. *N. sativa* is well known for its potent antioxidant effects.^24^

*N. sativa* oil (NSO) improved the anti‐inflammatory capacity of microglia while attenuating LPS‐induced inflammation, as shown in Table 1. By shifting the M1/M2 ratio toward the M2 state, NSO significantly reduced the inflammatory responses of LPS and improved the anti‐inflammatory status of microglia. These findings might support the idea that NSO functions as a potential immunomodulator for a number of neurodegenerative illnesses caused by M1 phenotype dominancy, including AD and PD.^25^

**Table 1:** Comprehensive summary of the neuroprotective effects of *N. sativa* and their potential nanoformulations.

Nano‐formulations	Disease	Results	Cell lines/animal models	References
NSO–pDNA–chitosan–PLGA‐based nanoparticles	Alzheimer's disease (AD)	The findings of this study suggest that NSO could be added to the gene delivery carrier to improve treatment benefits of AD	Neuro‐2A (N2a) cell line	[26]
Solid–lipid nanoparticle‐encapsulated thymoquinone (TQ‐SLNs)	Huntington's disease	The TQ‐SLNs (10 and 20 mg/kg)‐treated animals showed a significant (*p* < 0.01) improvement in muscle strength, rigidity, movement, and memory performances on the 7th and 14th day behavioral analyses	Wistar rats	[27]
Thymoquinone mucoadhesive nanoemulsion (TMNE)	Cerebral ischemia	After intranasal administration of TMNE, cerebral ischemic rats with middle cerebral artery occlusion demonstrated improved neurobehavioral activity (locomotor and grip strength)	Middle cerebral artery occlusion‐induced cerebral ischemic rats	[28]
Polysorbate‐80‐coated thymoquinone PLGA nanoparticles (P‐80‐TQN)	AD	TQ in P‐80‐TQN form rectified increased SOD levels and improved cognitive and behavioral dysfunctions in AD mice	Streptozotocin‐(STZ)‐induced Alzheimer's mice models	[29]
Thymoquinone‐rich fraction nanoemulsion (TQRFNE)	AD	The results demonstrated that TQRFNE's mechanistic actions in response to a high‐fat and high‐cholesterol diet are associated with Aβ generation, degradation, and transportation in rat brain tissue	High‐fat/cholesterol diet (HFCD)‐induced rats	[30]
Ropinirole hydrochloride (RHCl) nanoemulsion (NE) with nigella oil	Parkinson's disease	Experimental results showed that there was a high In vitro and a significant ex vivo nasal mucosal flux. In 6‐OHDA‐induced rats treated with drug‐loaded NE, Parkinson‐like symptoms were successfully reversed.	In vitro permeation using goat nasal mucosa and Albino Wistar rats were selected for In vivo studies	[31]
Thymoquinone solid–lipid nanoparticles (SLNs)	Depression via neuroprotective brain‐derived neurotrophic factor (BDNF) and downregulation of neuro‐inflammatory NF‐κB, IL‐6, and TNF‐α	TQSLN had significantly higher levels of neuroprotective BDNF (*p* < 0.010) as well as hippocampal 5HT/TRP. TQSLN may inhibit neuroinflammatory transmitters while favoring BDNF in the modulation of depressive neurobehavioral states.	LPS‐treated rats	[32]
TQ rich‐fraction nanoemulsion (TQRFNE); TQ emulsion; TQ nanoemulsion	Memory deficit; gene expression levels in the brain cortex and the hippocampus	Adding TQRFNE to a high‐fat, low‐cholesterol diet (HFCD) could improve the overall antioxidant status, antioxidant gene, and expression levels, alleviating memory deficits	Sprague–Dawley rats fed with a high‐fat‐cholesterol diet	[33]
PLGA–chitosan nanoparticles	Cerebral ischemia–reperfusion	Nose‐to‐brain administration of optimized TQ‐loaded PLGA NPs improved the pharmacokinetic profile of TQ in the brain, effectively reversing symptoms of cerebral ischemia and rescuing brain cells from ischemic death	Adult male Wistar rats	[34]
Thymoquinone‐loaded solid–lipid nanoparticles	Huntington's disease	By inhibiting microglial activation, NMDA receptor sensitization, and neuroinflammation, TQSLN (10 and 20 mg/kg) treatment has a promising beneficial effect on the main pathogenic characteristics of the 3‐NP model of HD	Albino male rats	[35]

Abbreviations: 3‐NP, 3‐nitropropionic acid; 5HT/TRP, 5‐hydroxytryptamine/L‐ tryptophan; 6‐OHDA, 6‐hydroxydopamine; Il‐6, interleukin 6; NMDA, *N*‐methyl‐d‐aspartate; NSO, *Nigella sativa* oil; PLGA, poly (lactic‐co‐glycolic acid); SOD, superoxide dismutase; TNF‐α, tumor necrosis factor α.

## 5 ROLE OF TQ AND NEURODEGENERATIVE

### 5.1 Diseases

NSO showed anticonvulsant properties in epileptic episodes in rats induced by electroshock. Recently, alpha‐amylase inhibitory activity of silver nanoparticles produced from NSO was revealed in an in vitro cell‐free experiment, confirming its hypoglycemic effects.^21^ Hepatoprotective, cancer‐fighting, immune‐modulating, anti‐inflammatory, antihypertensive, and antidiabetic effects are induced.^36^ Brain tissues are the most susceptible to oxidative damage due to the injury caused by ischemia–reperfusion, which produces a significant amount of ROS (hydroxyl radical, superoxide, and hydrogen peroxide). TQ, an antioxidant, reduces ROS‐mediated reactions by acting as a free radical scavenger or trapping agent. TQ protects brain cells from oxidative stress injury, which is more pronounced in the areas responsible for memory function. TQ works as an antiepileptic on the CNS as well. In AD, TQ reduces stress‐related inflammation and has been shown to protect neurons from A‐induced injury in the pheochromocytoma cell line (PC12 cell line). TQ has antineurotoxic properties, which contribute to its pharmacological characteristics, and it has been suggested that it may help to protect against NDs. TQ offers protection against some medications, primarily those with high toxic doses, like morphine and pentylenetetrazol. The aforementioned substances are potential risk factors for the development of neurodegenerative conditions that affect the CNS, including AD, PD, and dementia.^37^

## 6 TQ'S POTENTIAL FOR USE IN THE DELIVERY OF DRUGS FOR NEURODEGENERATIVE DISORDERS

Because current treatments for NDs have significant side effects such as headache and nausea, there is still a need to develop new strategies that are less harmful. Natural products appear promising in this regard, but their penetration through the BBB is a major barrier to their delivery to the nervous system. In this way, nanotechnology, and more specifically, nanomedicine or pharmaceutical nanotechnology, provide superior drug delivery systems for ND management through improved molecular monitoring, control, construction, repair, and diagnosis.^38^ The health of patients with NDs was found to be significantly improved with the use of a number of nanoformulations prepared from a variety of natural products, including curcumin, resveratrol, piperine, *Ginkgo biloba*, and *N. sativa*.

According to Farkhondeh and colleagues given TQ's neuropharmacological potential, which suggests that it could be a useful agent against neurological disorders, its formulations and delivery methods warrant further investigation. Delivery is crucial to clinical trials testing TQ's efficacy against neurodegenerative disorders. Because TQ has a hydrophobic chemical makeup, it dissolves poorly in water and has poor formulation properties. Due to its chemical makeup, it has a weak membrane penetration in humans.^39^ SLNs, TQ‐encapsulated chitosan NPs, TQ‐loaded liposomes, caryophyllene, and germacryl conjugates, as well as fatty acid conjugates and TQ‐loaded nanostructured lipid carriers, among other analogs of TQ, have been synthesized recently and may affect bioavailability and use in the clinical phase.^40^

Despite the fact that numerous studies have been carried out, further In vitro (Table 2), In vivo (Table 3), and In silico (Table 4) studies should still be carried out to develop more natural nanoformulations, like *N. sativa*, against neurodegenerative disorders like AD and PD.

**Table 2:** An extensive summary of black cumin's protective effects against neurodegenerative and mental issues through In vitro studies.

Objective	Cell lines	Neurodegenerative disease	Results	References
*N*. *sativa* for the treatment or the prevention of neurodegenerative diseases such as Parkinson's disease (PD)	PC12 cell line	PD	**↓** COX activity; **↑** MMP; attenuated MPP+‐mediated apoptosis	[41]
The antioxidant effects of TQ on microglia activated by the presence of LPS/IFN or H_2_O_2_	BV‐2 cell line	Microglia‐derived neurodegeneration	**↓** Superoxide and nitric oxide; **↓** hydrogen peroxide levels; **↓** oxidative stress; **↓** lipid hydroperoxides; **↑** antioxidant GSH; **↑** antioxidant glutathione; **↓** ROS activities; **↓** SOD and CAT activities	[42]
TQ on mitochondrial dysfunction and oxidative stress in differentiated PC‐12 cells exposed to A fragment 25–35 (A_25__–35_) was investigated	PC12 cell line	Alzheimer's disease (AD)	**↑** AChE activity; **↑** glutathione and its dependent enzymes	[43]
TQ's having antiapoptotic ability to reduce ‐amyloid peptide 1–40 sequence (A_1__–40_)‐induced neuronal cell death in primary cultured CGNs	Cerebellar granule neuron cell line	Neurotoxicity	Inhibition of Aβ_1–40_‐induced apoptosis; **↓** free radical generation; loss of condensed chromatin; and extensive neurite networks	[44]
To explore the protective potential of TQ in Aβ_1–42_‐induced neurotoxicity	Human iPSC‐derived cholinergic neuron cell line	AD	**↓** Synaptic activity; **↓** caspase 3/7; restored the content of GSH and significantly inhibited the apparent increase in H_2_O_2_	[45]
TQ's ability to inhibit neurodegeneration caused by MPP+ and MPTP In vivo and In vitro by acting as an activator of the Nrf2/ARE cascade	SH‐SY5Y cell line	PD	**↓** MPP+‐mediated cell death and apoptosis; **↑** nuclear translocation of Nrf2; **↑** expression of HO‐1, NQO1, and GST	[46]
The neuroprotective efficacy of TQ was explored by primarily studying its antioxidant and antiapoptotic potential against As_2_O_3_‐induced toxicity in SH‐SY5Y human neuroblastoma cell lines	SH‐SY5Y cell line	Neurotoxicity and As_2_O_3_‐induced cytotoxicity	**↑** Viability; **↓** free radical generation; repaired DNA; balanced transmembrane potential	[47]
The roles of cellular ROS, 50 AMP‐activated protein kinase (AMPK), and sirtuin 1 (SIRT1) in the antineuroinflammatory activity of TQ were investigated	BV2 mouse microglia cell line	Neuroinflammation	**↓** Cellular ROS generation; **↑** levels of LKB1 and phospho‐AMPK proteins; **↑** nuclear accumulation of SIRT1 protein; **↑** NAD^+^ protein levels; **↓** cytoplasmic levels	[48]

Abbreviations: As_2_O_3_, arsenic trioxide; CAT, catalase; COX, cyclooxygenase; GSH, glutathione; GST, glutathione‐*S*‐transferase; H_2_O_2_, hydrogen peroxide; HO‐1, heme oxygenase 1; IFNγ, interferon gamma recombinant mouse protein; iPSC, induced pluripotent stem cells; LKB1, liver kinase B1; MMP, mitochondrial membrane potential; MPP+, 1‐methyl‐4‐phenylpyridinium; MPTP, 1‐methyl‐4‐phenyl‐1,2,3,6‐tetrahydropyridine; PC12, pheochromocytoma; NAD^+^, nicotinamide adenine dinucleotide; NQO1, quinone oxidoreductase.

**Table 3:** An extensive summary of black cumin's protective effects against neurodegenerative and mental issues through In vivo studies.

Objective	Animal models	Neuro‐degenerative disease	Significant findings	References	
The impact of a hydroalcoholic extract of NSE and TQ was studied on rat cerebral hypoperfusion‐induced learning and memory deficits, hippocampal AChE activity, and markers of redox status, particularly lipid peroxidation and SOD activity	Male Wistar rats	Cerebrovascular insufficiency states and dementia	**↓** Lipid peroxidation levels; **↓** AChE activity; **↓** SOD levels; **↓** learning and memory impairments	[49]	
To test the effectiveness of TQ in the treatment of Parkinson's disease (PD), male Wistar rats were administered rotenone, which causes oxidative stress and affects mitochondrial dynamics such as fission and fusion	Male Wistar rats	PD	**↓** Oxidative stress; **↓**Drp1; **↑** Parkin; **↑** Dopamine; **↑** TH levels	[50]	
The study examined the effects of TQ on brain injury in a lithium‐pilocarpine rat model of status epilepticus and the underlying mechanism related to the antioxidative pathway	Male Sprague–Dawley (SD) rats	Seizures induced by lithium chloride and pilocarpine	**↑** Memory efficiency; **↓** NF‐κB; **↓** COX‐2; **↓** TNF‐α	[51]	
The purpose of this study was to determine the effect of TQ on learning and memory in a rat model of AD induced by a combination of AlCl_3_ and d‐Galactose	SD male albino rats	Alzheimer's disease (AD)	**↓** TNF‐α; **↓** IL‐1β; **↓** TLRs pathway components; **↓** NF‐κB and IRF‐3 mRNAs; **↓** Aβ formation and accumulation	[52]	
The aim of the study was to examine the protective and ameliorative effects of *N. sativa* seeds in Experimental autoimmune encephalomyelitis ‐induced Wistar rats	Adult female Wistar rats	Multiple sclerosis	Improved remyelination; **↓** inflammation; **↓** TGF β1	[53]	
The goal of the study was to determine whether TQ could reduce behavioral, cellular, and oxidative stress markers in a rat experimental model of early PD	Adult male Wistar rats	PD	**↓** MDA; **↓** loss of SNC neurons; improved turning behavior; neuroprotection against 6‐OHDA neurotoxicity	[54]	
Thymoquinone was evaluated in the study as a potential AD treatment in a transgenic *Drosophila melanogaster* model	*D. melanogaster*	AD	**↓** ROS production; **↓** gene and protein expression of hTau; **↑** behavioral activity; restoration of depleted SOD and AChE activities	[55]	
The aim of the experimental study was to examine the effects of a hydroalcoholic extract of *N. sativa* on markers of cerebral angiogenesis in rats induced by global brain ischemia	Male Wistar rats	Global ischemia of brain	**↓** Brain edema and infarct volume; **↑** gene expression of VEGF and HIF; **↓**matrix metallopeptidase‐9 activity	[56]	

Abbreviations: AlCl_3_, aluminum chloride; DRP1, dynamin‐related protein 1; HIF, hypoxia‐inducible factor; hTau, human Tau; IRF‐3, interferon regulatory factor 3; MDA, malondialdehyde; SNC, substantia nigra pars compacta; TGF β1, transforming growth factor beta 1; TH, tyrosine hydroxylase; VEGF, vascular endothelial growth factor.

**Table 4:** An extensive summary of black cumin's protective effects against neurodegenerative and mental issues through In silico studies.

Objective	Target	Neuro‐degenerative disease	Results	References
PHT (anticonvulsant) with TQ (neuroprotective) were combined, so as to prevent the neurodegenerative changes and enhance the clinical efficacy	Phenytoin against electroshock‐induced convulsions	Epilepsy	The computational studies revealed that PHT and TQ cooperatively bind the active site on Akt and establish a good network of intermolecular interactions, which indicates the sequential inhibition of PI3K/Akt/m‐TOR signaling with the combination	[57]
The study performed molecular docking, and formulated, and characterized TQ‐loaded SLN and showed comparative antidepressant activity	Crystal structure of LEUTAA	Depression	The results of the molecular docking study revealed that TQ showed greater affinity and tighter binding capacity for the active site of neurotransmitter receptors	[58]
The primary objective of the study was to test the effectivity of thymoquinone (TQ) in a Concanavalin A‐induced sickness behavior model of depression in mice	Concanavalin A‐induced behavior deficit	Inflammation‐related sickness behavior	Molecular docking analysis supported the notion that the antidepressant effect of TQ may be mediated by antagonism of SERT	[59]
Altogether, the mechanism of binding of TQ with hTf was elucidated, which can be further implicated in the development of a novel strategy for AD therapy	Binding mechanism of TQ to human transferrin	Alzheimer's disease	Molecular docking analysis showed key residues of the hTf that were involved in the binding to TQ. Further, a 250 ns molecular dynamics simulation was performed, which deciphered the dynamics and stability of the hTf–TQ complex.	[60]
SVP with BFN and TQ were combined to restrict seizures and provide adequate neuroprotection	The crystal structure of human Akt co‐crystallized with an inhibitor	Epilepsy	The Insilco results revealed that trio‐drug combination binds the Akt active site as a supramolecular complex, which could have served as a delivery system that affects the penetration and the binding to the new target	[61]

Abbreviations: AKT, protein kinase B; BFN, baclofen; Htf, human transferrin; mTOR, mammalian target of rapamycin; PHT, phenytoin; SERT, serotonin reuptake transporter; SVP, sodium valproate.

## 7 FUTURE PERSPECTIVES AND CONCLUSION

The prevalence of NDs is increasing with an increase in lifespan globally, necessitating novel treatment approaches to improve both symptoms and quality of life of patients with diseases such as Alzheimer's disease, Parkinson's disease, epilepsy, and dementia. Numerous studies have been conducted to determine the cause and potential therapeutic strategies for neurodegenerative damage, and many more are currently ongoing. Due to their potential therapeutic benefits and lack of negative side effects, many phytochemicals are of interest to researchers. The herb *N. sativa* is one of many. The current study demonstrated the neuroprotective benefits of *N. sativa* in the management of several NDs, including dementia, epilepsy, PD, and AD, which have been identified in numerous experiments. Even though there are some promising results for brain‐targeted drug delivery via invasive or noninvasive approaches using novel drug delivery systems for crossing the BBB, these are still not well defined. There is a need for more drug delivery systems that use natural formulations such as *N. sativa*, and more research is required. Researchers have recently made advancements in the development of NPs that inhibit cell surface chemicals involved in bruising and swelling. This treatment for AD is very safe due to the use of nanotechnology research. We believe that instructive studies pertaining to nano‐suspensions, nanoparticles, and nanoemulsions are required to explore this option further through research. The insufficient systemic availability of natural compounds for the treatment of ND is a major factor that restricts their therapeutic applications. Future research should be conducted to improve its systemic availability, resilience to metabolic reactions, and passage through the BBB using bio‐therapeutic technologies such as nano‐suspensions, NPs, and nanoemulsions.

## AUTHOR CONTRIBUTIONS

Chaitali G. Gawas collected the resources, was involved in the conceptualization of the study, and wrote the original draft. Sakshi Mathur contributed to editing and revision of the whole manuscript. Heena Tabassum contributed to the main ideas of this review, leading to the submission of the paper. Minal Wani finalized the review and approved the final version.

## CONFLICT OF INTEREST STATEMENT

The authors declare no conflict of interest.

## ETHICS STATEMENT

Not applicable.

## Data Availability

The data of our study are available on reasonable request.
